# Microsatellite Polymorphism and the Population Structure of Dugongs (*Dugong dugon*) in Thailand

**DOI:** 10.3390/ani12030235

**Published:** 2022-01-19

**Authors:** Anocha Poommouang, Promporn Piboon, Kittisak Buddhachat, Janine L. Brown, Wannapimol Kriangwanich, Siriwadee Chomdej, Jatupol Kampuansai, Supamit Mekchay, Patcharaporn Kaewmong, Kongkiat Kittiwattanawong, Korakot Nganvongpanit

**Affiliations:** 1 Department of Veterinary Biosciences and Public Health, Faculty of Veterinary Medicine, Chiang Mai University, Chiang Mai 50100, Thailand; anopotter@hotmail.com (A.P.); muy_v3@hotmail.com (P.P.); wannapimol.k@gmail.com (W.K.); 2 Excellence Center in Veterinary Bioscience, Chiang Mai 50100, Thailand; k_buddhachat@yahoo.com (K.B.); siriwadee@yahoo.com (S.C.); 3 Department of Biology, Faculty of Science, Naresuan University, Phitsanulok 65000, Thailand; 4 Smithsonian Conservation Biology Institute, Center for Species Survival, 1500 Remount Road, Front Royal, VA 22630, USA; brownjan@si.edu; 5 Department of Biology, Faculty of Science, Chiang Mai University, Chiang Mai 50200, Thailand; Jatupol_k@hotmail.com; 6 Department of Animal and Aquatic Sciences, Faculty of Agriculture, Chiang Mai University, Chiang Mai 50200, Thailand; supamit.m@cmu.ac.th; 7 Phuket Marine Biological Center, Phuket 83000, Thailand; marineanimal.vet@gmail.com (P.K.); kkongkiat@gmail.com (K.K.)

**Keywords:** DNA marker, population genetic, simple sequence repeat, Sirenia

## Abstract

**Simple Summary:**

For this study, skin samples were analyzed from 77 individual stranded dugongs collected in Thai waters from 1994–2019 using six microsatellite markers to assess the genetic diversity and population structure. Dugongs in the Andaman Sea had higher genetic variation than those in the Gulf of Thailand. Populations in Trang, Satun and some areas of Krabi had highest diversity compared to other regions of Thailand. The analysis of Bayesian genetic clustering showed that dugongs in Thailand consist of five genetic groups. Furthermore, dugongs in the middle and lower Andaman Sea presented the greatest gene flow compared to other regions. Based on calculation of inbreeding coefficients, dugong populations in the Sea of Thailand are experiencing some levels of inbreeding, and so may warrant special protections. Results of this study provide important information on genetic diversity and genetic population structuring of dugongs in Thailand and for understanding the genetic status of dugongs that can lead to improved management and conservation of this endangered species.

**Abstract:**

The dugong (*Dugong dugon*) is an endangered species of marine mammals, so knowledge of genetic diversity of these populations is important for conservation planning within different habitats. In this study, six microsatellite markers were used to assess the genetic diversity and population structure of 77 dugongs from skin samples of stranded animals collected from 1994–2019 (69 from Andaman Sea and 8 from the Gulf of Thailand). Our results found that dugongs in the Andaman Sea had higher genetic variation than those in the Gulf of Thailand. Populations in Trang, Satun, and some areas of Krabi had highest diversity compared to other regions of Thailand. Bayesian genetic clustering analysis revealed that dugongs in Thailand consist of five genetic groups. Moreover, dugongs in the middle and lower Andaman Sea presented the greatest gene flow compared to other regions. However, based on calculation of inbreeding coefficients (Fis value = 0.239), dugong populations in the Sea of Thailand are experiencing some levels of inbreeding, and so may warrant special protections. These results provide important information for understanding the genetic status of dugongs that can lead to improved management and conservation of this endangered species.

## 1. Introduction

The dugong (*Dugong dugon*), order Sirenia, family Dugongidae, is the only herbivorous marine mammal in the world, with a diet consisting of seagrass [1]. Dugongs have a life span of around 60–70 years [2], sexual maturity at ~10–12 years of age [3], and reach adulthood at 20 years of age [4]. Seagrass is the main food source of dugongs [5,6,7,8], so degradation and disappearance of these meadows are major reasons for declining dugong populations. Most of seagrass degradation is due to coastal construction, pollution, and illegal fishing [9]. It is also affected by seasonal changes in some areas [10]. The dugong feeds on bottom-dwelling, emergent, and riparian aquatic plants in the tropics and subtropics [11,12,13]. Dugong reproductive behaviour has been minimally described due to the difficulty of observing them in the wild, but in general they live, mate, and nurse offspring in seagrass areas [11,12,13]. In high density areas like Australia, dugongs exhibit a large range of individualistic movement behaviors, with some being relatively sedentary, while others migrate over hundreds of kilometers, sequentially grazing seagrass meadows to prevent overgrazing [14]. In other parts of their range, including southeast Asia, where populations have undergone drastic declines, dugongs travel in smaller groups and can show high site fidelity to a few meadows that support limited numbers of individuals [11]. Large-scale movements in Sirenians also occur in response to seasonal changes in environmental variables, such as temperature, water levels, salinity, and variability of forage [15].

The dugong is native to coastal waters of the Indo-Pacific Ocean, and listed as vulnerable to extinction by International Union for Conservation of Nature (ICUN) and Natural Resources [16], and in the Convention on International Trade in Endangered Species of Wilde Fauna and Flora (CITES Appendix I) due to population declines and degradation of habitat. For example, in Australia, the estimated rate of decline averaged about 8.7% per year between 1962 and 1999, resulting in a 97% reduction in initial catch rates over a 38-year period [17]. The largest populations today are found along the coasts of Australia (~10,000 dugongs) [18], followed by the Arabian/Persian Gulf (~6000) [19,20], Red Sea (~2000) [6], New Caledonia (~898) [18,21], and Mozambique (~300) [6]. Dugongs have completely disappeared from areas around Japan (Sakishima Shoto islands), Hong Kong, Maldives, Mauritius, Philippines, Taiwan, Cambodia, and Vietnam. 

In Thailand, dugongs were once common along both coasts of the Andaman Sea and Gulf of Thailand, but have since declined severely [22]. Approximately 200 dugongs were estimated to live in the Andaman Sea region [23], with aerial surveys in 1997, 1999, 2000, and 2001 finding only small populations from Ranong to Satun Provinces. The largest viable population was in Trang Province [24,25], estimated at 120 individuals, which likely is the largest and healthiest group of dugongs in Southeast and Eastern Asia [23,26]. Smaller numbers persisted in eastern Thailand near the border with Cambodia and throughout the Gulf of Thailand [27], with a total population estimate of 50 individuals [24,28]. According to a more recent survey by the Phuket Marine Biological Center in 2017 [29], the number of dugongs in Thailand waters was 221. That survey found 191 dugongs (86%) lived in the Andaman Sea with 30 (14%) in the Gulf of Thailand, mostly in non-hunting areas where seagrass is still intact. In 2016, a total of 12 dugongs were stranded in the Andaman Sea [29], which was higher than that between 2011–2015 [29]. Most stranded dugongs were deceased (83%), with the majority of causes (74–89%) attributed to fishing gear [29]. Severe population declines of dugongs throughout their range in Southeast Asia are a concern, and since 2010 have been under the protection of the Wild Animal Reservation and Protection Act, B.E.2553 [25], with regional threats abounding.

Examining population structure is an important aspect of evolutionary genetics and such studies are of vital importance for conservation and management. In the past decades, researchers have studied the genetic diversity of several Sirenia in diverse habitats using different techniques. For example, Seddon et al. [30] used microsatellite markers and found genetic diversity was low in dugongs in southern Queensland, Australia. A significant population structure was detected and mean pairwise relatedness values within populations were low as well. Using mitochondrial DNA (mtDNA) sequences, Plon et al. [31] found a 355 bp sequence in the D-loop that matched dugongs from Australia and Indonesia, and revealed several new and divergent mtDNA lineages in the Indian Ocean. Recently, a study from our group reported a unique genetic structure in dugongs found only in the Andaman Sea of Thailand [32]. In this study, tissue samples from dugongs stranded off the coast of Thailand during 1994–2019 were analyzed using microsatellite markers to provide more information on genetic diversity and population structuring in the region. 

## 2. Materials and Methods

### 2.1. Sample 

Samples (skin tissue) from 77 deceased dugongs (male = 36, female = 41) that were stranded between 1994–2019 (Figure S1, Table S1) were provided by the Phuket Marine Biological Center, Phuket, Thailand. The samples were collected and preserved in 95% ethanol at −20 °C. Use of banked samples meant animal ethics committee approvals were not required.

### 2.2. DNA Extraction

The samples were processed using DNA extraction kits according to manufacturer’s instructions (DNeasy Blood & Tissue Kit, Qiagen, Germany) at the Faculty of Veterinary Medicine, Chiang Mai University [32], and the DNA measured qualitatively and quantitatively by agarose gel electrophoresis and spectrophotometry, respectively [4].

### 2.3. Microsatellite Amplification and Genotyping

Twelve microsatellitess were selected from previously published studies [33,34,35,36,37] and screened twice using a polymerase chain reaction (PCR) technique resulting in the selection of six that produced reproducible and unambiguous bands (Table 1). Three individuals were amplified individually for screening by PCR with 1X ViBuffer S (16 mM (NH_2_)_4_SO_4_, 50 mM Tris-HCl, 1.75 mM MgCl_2_, and 0.01% TritonTM X-100), 0.2 µM dNTP (Vivantis, Selangor Darul Ehsan, Malaysia), 0.2 µM microsatellite primer (each forward primers had a 5′ M13 complementary tail to enable labeling with a fluorescent M13 primer [38]), 1 U Taq DNA polymerase (Vivantis, Selangor Darul Ehsan, Malaysia), and 10 ng DNA template with deionized water added to a volume of 25 µL. In each PCR reaction, deionized water was used as a negative control. PCR amplifications were performed in PTC-200 at DNA EngineThermal Cycler (Bio-Rad Laboratories, Inc., CA, USA) under the following conditions: 95 °C for 5 min, followed by 40 cycles of 95 °C for 30 s, 50 °C for 45 s, and 72 °C for 1 min with a final extension step at 72 °C for 10 min. The PCR products were stained [32] by REDSAFE Nucleic acid staining solution (iNtRON Biotechnology, Gyeonggi-do, South Korea) and then separated electrophoretically on 2% agarose gel (PanReac AppliChem ITW companies, Darmstadt, Germany) by PowerPac 200 (Bio-Rad, Hercules, CA, USA) containing 1X Tris-acetate-ethylenediaminetetraacetate (TAE) buffer at 120 V for 30 min. The PCR products were then visualized by UV light under a GELMAX 125Imager (UVP, Cambridge, England) [32]. A fragment analysis platform was used (3730XL-96 genetic analyzer; Thermo Fisher Scientific, Foster, CA, USA), performed by Ward Medic Ltd. Vadhana, BKK, Thailand. The fragments were manipulated and sized using the program GENE MARKER version 2.6.2 [39].

### 2.4. Statistical Analysis

#### 2.4.1. Microsatellite Analysis

**Figure 1:**
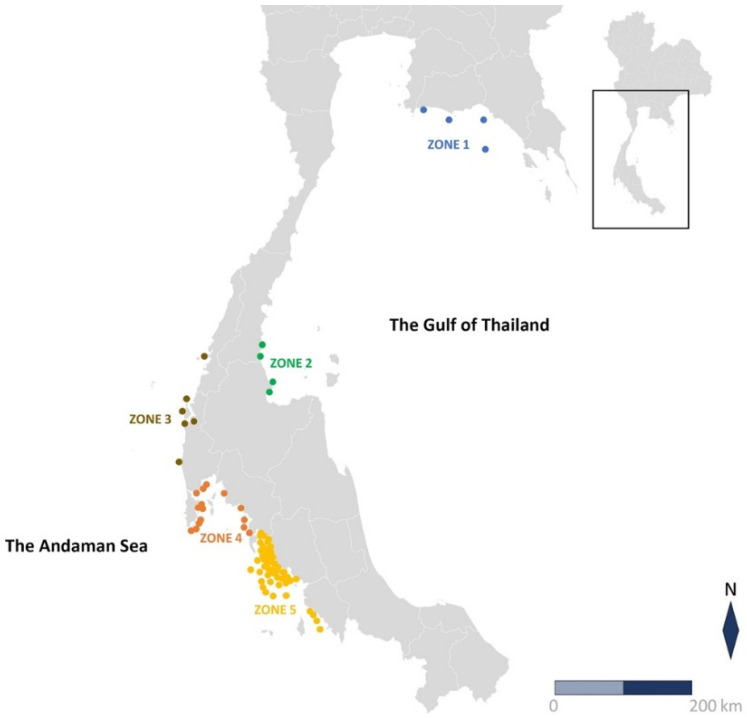
Geographic map of the Sea of Thailand. Location of 77 sample collections: Zone 1, upper the Gulf of Thailand (*n* = 4); Zone 2, lower Gulf of Thailand (*n* = 4); Zone 3, upper Andaman Sea (*n* = 6); Zone 4, middle Andaman Sea (*n* = 15); and Zone 5, lower Andaman Sea (*n* = 48).

Microsatellite comparisons were made among five location zones (Figure 1): upper Gulf of Thailand (Zone 1), lower Gulf of Thailand (Zone 2), upper Andaman Sea (Zone 3), middle Andaman Sea (Zone 4), and lower Andaman Sea (Zone 5). These zones are based on data from the Central Database System and Data Standard for Marine and Coastal Resources, Thailand (http://km.dmcr.go.th (accessed on 7 August 2020)), which identified discrete clusters of dugongs from aerial surveys of living dugongs in the seas of Thailand. Microsatellite allele frequencies were calculated by a simple counting scheme. The genetic diversity indexes [40,41] including Hardy–Weinberg Equilibrium *p*-value (HWE *p*-value), observed number of alleles (Na), effective number of alleles (Ne), Shannon’s information index (I), observed heterozygosity (Ho), expected heterozygosity (He), F-statistics, and Nei’s genetic distance were analyzed by GENALEX program version 6.5 [42]. These values were used to determine genetic variation, with higher values indicating higher genetic diversity. In addition, the smaller the genetic distance value, the more genetically similar the groups are. All alleles in all locations for all populations were analyzed by GENALEX program version 6 [43]. The genotype was adjusted and the Hardy–Weinberg Equilibrium was retested at the locations where null alleles were detected. The statistical parameters of forensic interest, including power of discrimination (PD), matching probability (MP) and power of exclusion (PE) were calculated by GENALEX program version 6 [43].

#### 2.4.2. Population Structure

A total of 77 samples were analyzed by distance and model-based clustering methods to reveal population affinity and structure. Pairwise linearized genetic distance based on the difference in allele frequencies (Fst) was computed by GENALEX program version 6.5 [42]. The program STRUCTURE version 2.3.4 [44,45] was used to cluster individuals into populations on the basis of microsatellite genotypes with admixture assumed and correlated allele frequencies [44,45]. The LOCPRIOR model was used to infer cryptic population structure [46]. Three runs for each number of 1 to 10 clusters (K value) were carried out with a Markov chain Monte Carlo (MCMC) chain burn-in length of 100,000 iterations and a 1,000,000 iteration run length. Chain convergence was assessed through a comparison of the results acquired from three different chains. The ΔK statistics determined from subsequent K value were plotted by the STRUCTURE HARVESTER to identify the optimal number of clusters in the data. The proportion of genetic clustering in each region was taken into consideration for gene flow. Outputs from the STRUCTURE HARVESTER [47,48,49,50] were graphically modified by DISTRUCT [51]. The pairwise Fst comparison confirmed the genetic difference among populations. To visualize relationships among the population, a principal component analysis (PCA) plot was constructed from a distance matrix of linearized Fst by R-STUDIO program version 4.1 [52].

#### 2.4.3. Kinship Analysis

The ML-RELATE program [53] was used to calculate the maximum likelihood estimates of relatedness (r value) [54] as described by Wagner et al. (2006) [55]. ML-RELATE uses a downhill simplex routine to find the maximum likelihood estimate of r value. ML-RELATE estimates r for one pair of individuals only. The r value indicates a relatedness with a value between 0–1 (1 = strongly correlated, 0 = not correlated) with 0.05 level of significance. This method was chosen because maximum likelihood estimates of relatedness usually are more accurate than other estimators [56]. Relatedness analysis produced results in both matrix and list output formats. Heat map and clustering based on matrix output were produced by R-STUDIO program version 4.1 [52].

## 3. Results

### 3.1. Microsatellites

Allele frequency distributions and statistical parameters of forensic interest for each population are provided in Table S2. The number of samples that were amplified (and as a percent of the total number), range size, number of alleles, effective number of alleles, Shannon’s information index, observed heterozygosity, and expected heterozygosity for each microsatellite locus are shown in Table S3. The combined power of discrimination (PD) and power of exclusion (PE) was greater than 0.999 and 0.99, consecutively for all spatial populations (Table 2).

### 3.2. Population Structure

**Figure 2:**
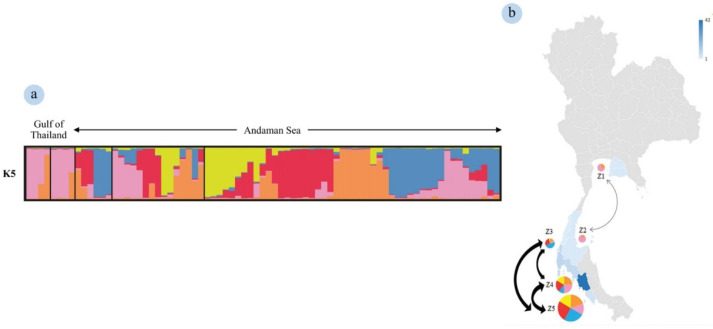
Admixture bar plot estimation figures of the dataset, with sequential delta K. The population structure consists of five genetic clusters indicated by different colors: orange, pink, blue, red, and yellow (**a**). Each individual is represented by a thin vertical line, which is partitioned into colored segments that represent the individual’s estimated membership fractions in the K5 (**a**). On the K5 plot, each column framed areas isolate each population such as defined on the map (**b**). The color bar and the regions in blue re represent the number of individuals sampled in each zone. The arrow shows the trend of gene flow within the Andaman Sea and Gulf of Thailand using data from the structure.

The population structure consisted of five genetic clusters (ΔK = 5), depicted as orange, pink, blue, red and yellow (Figure 2). In both upper and lower Gulf of Thailand, there were two dominant genetic clusters that were clearly visible, depicted as orange and pink. All three zones of the Andaman Sea had similar proportions of five genetic clusters, except in Zone 3 (lowest = yellow; greatest = blue). The gene flow between Zones 4 and 5 was higher than for Zones 3 to 4 or 5. However, there also was gene flow observed between Zones 1 and 2 (Figure 2b). 

**Figure 3:**
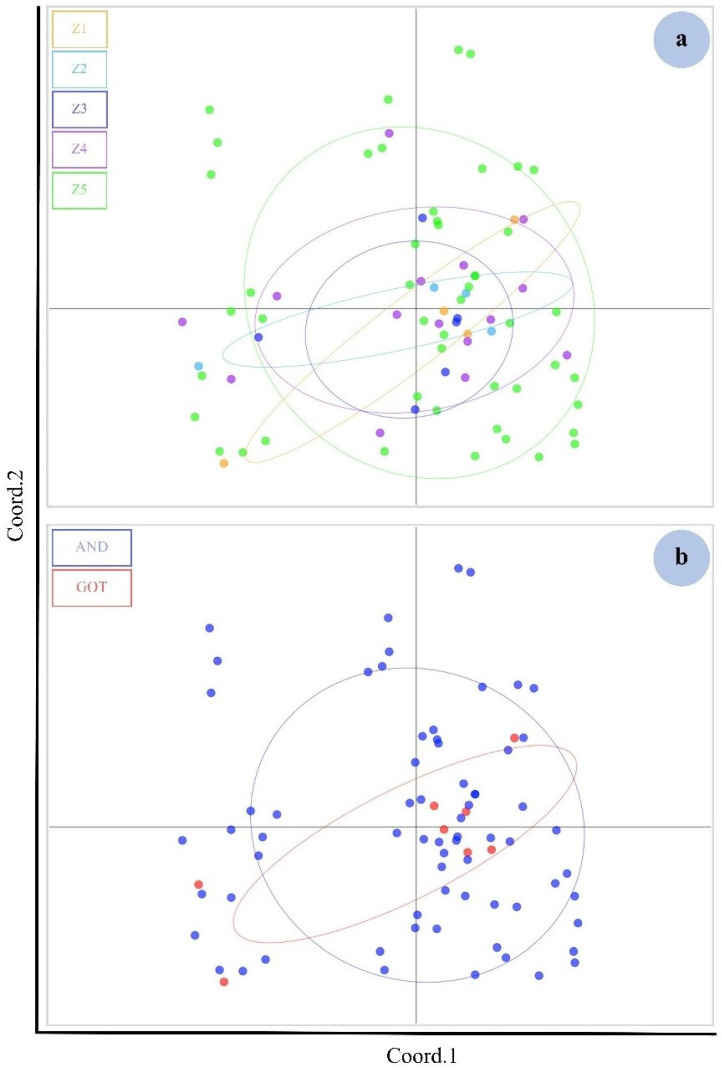
PCA plot (autosomal microsatellite data) showing the five indistinguishable genetic clusters by zones (Z) of dugongs with the principal components 1 (Coord.1) (12.70%) and principal components 2 (Coord.2) (10.47%) (**a**). The populations were divided into two groups: the Gulf of Thailand (GOT) and the Andaman Sea (AND) (**b**). Genetic characteristics could not be separated with the Coord.1 (12.70%) and Coord.2 (10.47%) (**b**).

The PCA of microsatellite loci of the five zones plotted on two axes cumulatively explained 35.41% of the variation (12.70% and 23.17%, respectively). The PCA plot revealed that the five zones were not clearly differentiated from each other (Figure 3a). The same value was found when the populations were divided into two groups (Figure 3b).

### 3.3. Kinship Analysis

**Figure 4:**
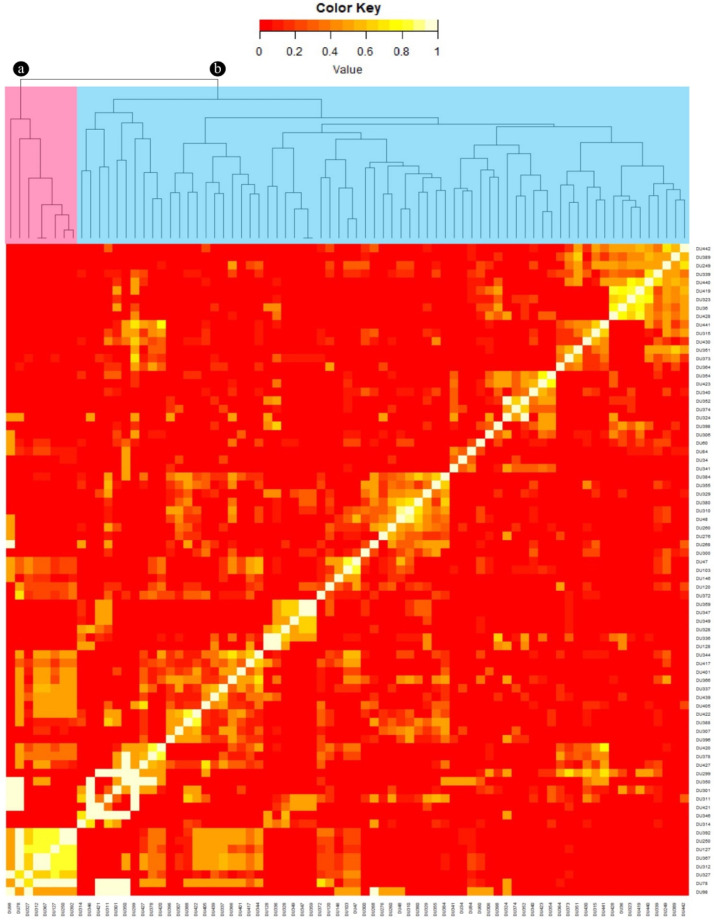
Kinship relatedness among dugong populations. Samples were categorized into two groups: the Gulf of Thailand (*n* = 8) labeled in pink (a); and the Andaman Sea (*n* = 69) labeled in blue (b).

Two genetically related groups of dugongs were identified: those from the Gulf of Thailand; and those from Andaman Sea (Figure 4). Those in the Andaman Sea could be categorized into five subgroups, while in the Gulf of Thailand, only one group was clearly separated from the others. 

### 3.4. Genetic Diversity

Genetic variation of dugongs in Zone 2 was the lowest compared to all other zones, with that in Zone 5 being the highest (Table 3). The range in observed heterozygosity was 0.26–0.45 and expected heterozygosity was 0.42–0.67 (Table 4). In all zones, all microsatellite loci deviated from the Hardy–Weinberg Equilibrium, with the exception of DduB02. When considering zones, locus Tmakb60 and Tma-FWC17 did not indicate differences between Zones 1 and 2 (Table 4), where significant (*p* < 0.05) deficiencies in heterozygotes were identified. The mean allele difference between the populations (Fst) was 0.138 and the mean number of migrants (Nm) was 2.944 (Table 4). The sum Nm values of Zones 1 and 2, 3 and 4, 3 and 5, and 4 and 5 were 1.37 ± 0.65, 12.74 ± 6.44, 9.88 ± 2.76 and 15.65 ± 3.74, respectively.

### 3.5. Genetic Differentiation

The pairwise Nei’s genetic distance analysis showed the lowest distance between Zones 4 and 5 in the Andaman Sea (0.06), and the highest between Zones 1 and 2 in the Gulf of Thailand (0.55) (Table 5).

## 4. Discussion

From this study, dugongs from the Andaman Sea were shown to have higher genetic diversity than those in the Gulf of Thailand, similar to data from previous inter-simple sequence repeat (ISSR) [32] and microsatellite [57] studies. In 2013, the first report of microsatellite markers (60 dugongs in the sea of Thailand between 1982–2007) found animals in the northern Andaman Sea had more genetic diversity than those in the Gulf of Thailand. In our study, we found the dugong population in Trang, Satun, and some areas of Krabi (Zone 5) had the highest genetic diversity, which also agrees with our previous work [32]. We found that the genetic clusters of dugongs living in the Gulf of Thailand were similar to those living in the Andaman Sea. But from the kinship analysis, dugongs living in seas on both sides of the country were completely separate. A previous microsatellite analysis [57] provided evidence of seven genetic clusters in the Thai dugong population. In the Gulf of Thailand, one genetic cluster dominated above all others, while in the Andaman Sea, similar groups were found, with few proportions compared to other clusters. This finding is similar to our present study, except we found only five clusters. Our previous study used a mitochondrial D-loop marker [32] and found that the genetic haplotype of dugongs in the Gulf of Thailand differed from that in the Andaman Sea. Taken together, these studies provide strong evidence that dugongs living in the Andaman Sea are genetically differently from those living in the Gulf of Thailand, and could present biological differences within each habitat. Finally, a study in 2017 [58] reported significant differences in skull and scapular morphology between dugongs in the Gulf of Thailand and Andaman Sea, further suggesting genetic differences between these populations, although confirmatory studies are needed.

We found that dugongs in the Gulf of Thailand and Andaman Sea have different population structures. Because there is no movement between the Gulf of Thailand and Andaman Sea [32], and they normally inhabit shallow shores near seagrass habitats [6], dugongs in the Gulf of Thailand and Andaman Sea area have their own genetic structures. When population structure was examined for each genetic clustering, no kinship relationships were found. Therefore, the difference in genetic clustering would be due to genetic differences of each dugong; those belonging to the same genetic clustering group were not from the same family. Looking at genetic migration, there were no differences between zones of the Gulf of Thailand, while in the Andaman Sea, the degree of migration was higher between Zones 4 and 5 compared to Zones 3, 4 or 5. Dugongs in areas around Trang Satun and some areas of Krabi had the highest genetic diversity, which might be related to those being the main dugong habitats; dugong numbers in those areas were the highest compared to other areas (Phuket Marine Biological Center) [29]. Seagrass areas in Thailand cover 255 square kilometers, distributed along the coast of six provinces in the Andaman Sea: Ranong, Phang-nga, Phuket, Krabi, Trang, and Satun. In the Gulf of Thailand, seagrass is found in 13 provinces, including Trat, Chanthaburi, Rayong, Chonburi, Phetchaburi, Prachuap Khiri Khan, Chumphon, Surat Thani, Nakhon Si Thammarat, Phatthalung, Songkhla, Pattani, and Narathiwat [29,59]. Of the 60 species of seagrass in the world [60], 13 are found in the Andaman Sea, and 12 in the Gulf of Thailand [7]. The seagrass area in the Andaman Sea is larger than that in the Gulf of Thailand [9], perhaps because wastewater discharge into the Andaman Sea contains only domestic waste, whereas in the Gulf of Thailand, it consists of both domestic waste and more toxic waste from industrial factories [61]. Future studies should examine how site fidelity among dugong populations might help explain some of the genetic clustering observations.

A study from Bushell (2013) [57] reported the inbreeding coefficient was low (Fis value of 0.055) among three subpopulations of dugong in the seas of Thailand (Gulf of Thailand and Andaman Sea) over a 27-year period between 1982 to 2008, indicating it was unlikely there was significant inbreeding. However, in our study of dugongs evaluated over a 26-year period, a higher level of inbreeding was observed based on the inbreeding coefficient (Fis = 0.24, Fst = 0.14, Fit = 0.32). When or how this inbreeding occurred is not known, but it suggests it may be recent, perhaps related to continuing degradation of seagrass habitat with more fragmentation and isolation of population. Given that smaller populations can show high site fidelity to meadows capable of supporting them without degradation [11], it is important to examine how this is related to genetic clustering, and if that is a cause of inbreeding at present. If so, future studies should monitor reproduction and mortality, and determine if reductions in genetic variability are affecting overall levels of population fitness. This is particularly important for dugongs in the Andaman Sea that have less genetic diversity, which could lead to faster extinction. These data are important because they suggest that dugongs and the seagrass meadows they depend on may require special government protections to prevent further population declines and decreases in genetic diversity. Ultimately, saving dugong populations will require a number of fundamental changes that potentially require government interventions. For example, finding ways to conserve seagrass and restore degraded seagrass meadows is of the utmost importance to ensure animals have enough food to live and reproduce, thus sustaining their numbers. Governmental regulations also are needed to control industrial and community waste through improvements in water sewage treatment systems before it is released into the sea. Finally, fisheries should refrain from using equipment and netting that results in accidental drownings and injuries.

This study had some limitations. First, the number of samples was not equal across the zones due to differences in stranding rates and sample availability. Second, there were no samples available between 2003–2008 because they were used for other research projects. Third, success was accomplished using only six microsatellite markers. From our study, the MP value was 0.000000226, meaning that over 10 million dugongs had the same genetic makeup as two dugongs; however, the dugong population in Thai seas is considerably less than 10 million animals. Thus, six microsatellites markers were enough to evaluate the genetic diversity of these dugong populations.

## 5. Conclusions

This study found that dugongs throughout the Thai seas have a similar genetic structure, although with different proportions. In the Gulf of Thailand, two dominant genetic structures were identified, while in the Andaman Sea, all five genetic structures were found in similar proportions. Moreover, the gene flow was similar between zones on the same coasts, with the greatest flow found between the middle and lower Andaman regions. In addition, our data found that dugong populations are exhibiting signs of inbreeding. The information from this study revealed the genetic status of dugongs in Thailand, which should be useful in developing management and conservation guidelines for the species in the long-term. At present, captive breeding has not been successful in dugongs, thus limiting its effectiveness as a conservation strategy for the species. Therefore, it is important to conserve extant wild populations through legal regulations to stop the continuous decline in both the Andaman Sea and the Gulf of Thailand.

## Figures and Tables

**Table 1:** Nucleotide sequences of microsatellites use in this study.

Locus	Sequence (5′-3′)	Fluorescent Dye	Length (bp)	References
TmaA04	(CT)_2_(GT)_12_AT(GT)_7_AT(GT)_2_	-	195–227	Garcia-Rodriguez et al., 2000 [34]
Tmakb60	(TG)_4_(CG)_1_(TG)_12_(CG)_6_	FAM	222–238	Pause et al., 2007 [36]
Tma-FWC03	(CTG)_6_TT(CTG)_4_TT(CTG)_7_	-	146–150	Tringali et al., 2008 [37]
Tma-FWC04	(AC)_12_(ATTT)_4_	FAM	175–211	Tringali et al., 2008 [37]
Tma-FWC08	(AC)_13_	-	149–159	Tringali et al., 2008 [37]
Tma-FWC11	(ca)_17_	-	123–127	Tringali et al., 2008 [37]
Tma-FWC17	(GT)_18_	FAM	201–209	Tringali et al., 2008 [37]
DduB01	(TG)_33_	FAM	332–374	Broderick et al., 2007 [33]
DduB02	(TG)_33_	ROX	198–224	Broderick et al., 2007 [33]
DduE04	(CA)_28_	-	324–338	Hunter et al., 2009 [35]
DduC05	(CA)_27_	HEX	218–230	Broderick et al., 2007 [33]
DduG12	(TG)_27_	-	378–406	Hunter et al., 2009 [35]

**Table 2:** Statistical parameters of dugong zone in the Gulf of Thailand and Andaman Sea based on six microsatellite loci across five regional zones.

Zone	PD	PE	MP
Zone 1 (*n* = 4)	0.999	0.991	0.0002
Zone 2 (*n* = 4)	0.999	0.991	0.0005
Zone 3 (*n* = 6)	0.999	0.995	0.00006
Zone 4 (*n* = 15)	0.999	0.999	0.0000006
Zone 5 (*n* = 48)	0.999	0.999	0.0000003
Overall	0.999	0.999	0.0000002

PD = power of discrimination; PE = power of exclusion; MP = matching probability.

**Table 3:** Diversity index values (mean ± SE) for dugongs in the five regional zones in Thailand.

Zone	Na	Ne	I	Ho	He
Zone 1 (*n* = 4)	3.33 ± 0.92	3.06 ± 0.91	0.88 ± 0.34	0.36 ± 0.16	0.43 ± 0.16
Zone 2 (*n* = 4)	3.00 ± 0.82	2.70 ± 0.76	0.80 ± 0.31	0.26 ± 0.16	0.42 ± 0.16
Zone 3 (*n* = 6)	4.17 ± 0.98	3.19 ± 0.94	1.07 ± 0.28	0.42 ± 0.14	0.53 ± 0.12
Zone 4 (*n* = 15)	8.00 ± 2.11	5.56 ± 1.75	1.53 ± 0.39	0.44 ± 0.11	0.62 ± 0.14
Zone 5 (*n* = 48)	10.17 ± 2.44	5.94 ± 2.00	1.64 ± 0.36	0.45 ± 0.13	0.67 ± 0.10

Na = observed number of alleles; Ne = effective number of alleles; I = Shannon’s information index; Ho = observed heterozygosity; He = expected heterozygosity.

**Table 4:** Summary of F-coefficients (differentiation between zones) and tests for Hardy–Weinberg Equilibrium (HWE) of six microsatellites in Thailand dugongs.

Locus	Fis	Fit	Fst	Nm	HWE *p*-Value					
					Zone 1	Zone 2	Zone 3	Zone 4	Zone 5	All
DduB01	0.15	0.23	0.10	2.27	0.28 ^ns^	0.17 ^ns^	0.68 ^ns^	0.09 ^ns^	0.00 *	0.00 *
Tmakb60	0.58	0.78	0.46	0.29	Monomorphic	Monomorphic	0.05 *	0.24 ^ns^	0.00 *	0.00 *
Tma-FWC17	0.17	0.24	0.08	2.83	Monomorphic	Monomorphic	0.82 ^ns^	0.99 ^ns^	0.00 *	0.00 *
DduCO5	−0.06	0.02	0.08	3.01	0.45 ^ns^	0.17 ^ns^	0.13 ^ns^	0.06 ^ns^	0.00 *	0.00 *
Tma-FWC04	0.74	0.76	0.07	3.45	0.10 ^ns^	0.06 ^ns^	0.21 ^ns^	0.00 *	0.00 *	0.00 *
DduB02	−0.15	−0.10	0.04	5.82	0.77 ^ns^	0.77 ^ns^	0.62 ^ns^	0.95 ^ns^	0.97 ^ns^	0.97 ^ns^
MeanSE	0.24 ± 0.14	0.32 ± 0.15	0.14 ± 0.07	2.94 ± 0.73						

ns = not significant, * *p* < 0.05.

**Table 5:** Pairwise population matrix of Nei’s genetic distance (metric below the diagonal, light grey box) and Fst values (metric above the diagonal, dark grey box) for dugongs in the five zones.

Zone 1	Zone 2	Zone 3	Zone 4	Zone 5	
0.00	0.25	0.07	0.06	0.08	Zone 1
0.546	0.00	0.173	0.10	0.14	Zone 2
0.169	0.53	0.00	0.04	0.03	Zone 3
0.116	0.29	0.13	0.00	0.02	Zone 4
0.137	0.44	0.10	0.06	0.00	Zone 5

## Data Availability

All relevant data are within the paper and its Supporting information files.

## Supplementary Materials

The following are available online at https://www.mdpi.com/article/10.3390/ani12030235/s1, Figure S1. Number of samples in this study. Table S1: Information of animal use in this study. Table S2 Allele frequency distributions and statistical parameters of forensic interest for each population. Table S3. Microsatellite polymorphism in dugongs in both the Gulf of Thailand and Andaman Sea.
