# Supplementary material for: Microsatellite Polymorphism and the Population Structure of Dugongs (Dugong dugon) in Thailand
Source: Animals (Basel). 2022 Jan 19;12(3):235. doi: 10.3390/ani12030235 (PMC8833502; doi:10.3390/ani12030235)
Supplement: Supplementary file 1 [file animals-12-00235-s001.zip › Figure S1.pdf]

Microsatellite polymorphism and the population structure of dugongs (*Dugong dugon*) in Thailand.

Anocha Poommouang, Promporn Piboon, Kittisak Buddhachat, Janine L. Brown, Wannapimol Kriangwanich, Siriwadee Chomdej, Jatupol Kampuansai, Supamit Mekchay, Patcharaporn Kaewmong, Kongkiat Kittiwattanawong, and Korakot Nganvongpanit

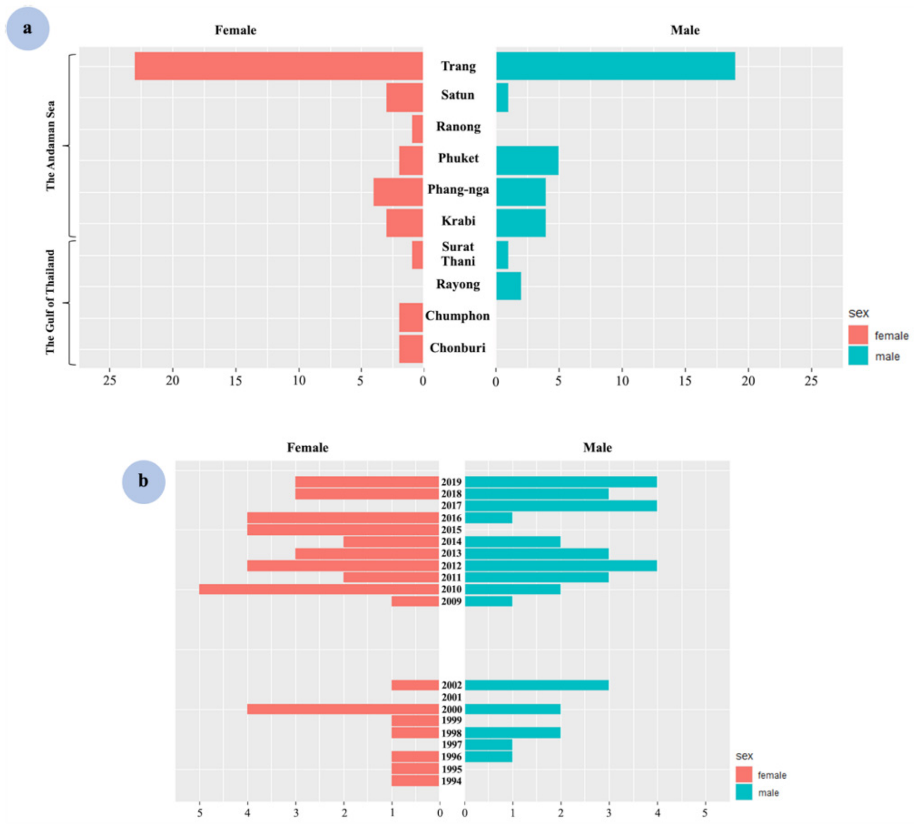

Figure S1. Number of samples in this study. Numbers of skin samples from male and female dugongs collected in 10 provinces in the Andaman Sea and Gulf of Thailand (a) and the years that skin sample were collected from 1994 to 2019 (b).
