# Supplementary material for: Microsatellite Polymorphism and the Population Structure of Dugongs (Dugong dugon) in Thailand
Source: Animals (Basel). 2022 Jan 19;12(3):235. doi: 10.3390/ani12030235 (PMC8833502; doi:10.3390/ani12030235)
Supplement: Supplementary file 1 [file animals-12-00235-s001.zip › Table S3 Microsatellite polymorphism and the population structure of dugongs.pdf]

Microsatellite polymorphism and the population structure of dugongs (*Dugong dugon*) in Thailand.

Anocha Poommouang, Promporn Piboon, Kittisak Buddhachat, Janine L. Brown, Wannapimol Kriangwanich, Siriwadee Chomdej, Jatupol Kampuansai, Supamit Mekchay, Patcharaporn Kaewmong, Kongkiat Kittiwattanawong, and Korakot Nganvongpanit

Table S3. Microsatellite polymorphism in dugongs in both the Gulf of Thailand and Andaman Sea.

Locus	Genetic variabilities	Andaman Sea (AND)	Gulf of Thailand (GOT)
Tmakb60	N	41 (59.420%)	2 (25.000%)
	Size range (bp)	238-262	242
	Na	7.000	1.000
	Ne	2.309	1.000
	I	1.138	0.000
	Ho	0.146	0.000
	He	0.567	0.000
Tma-FWC04	N	61 (88.406%)	8 (100%)
	Size range (bp)	171-191	173-183
	Na	12.000	3.000
	Ne	6.834	2.462
	I	2.114	0.974
	Ho	0.246	1.000
	He	0.854	0.594
Tma-FWC17	N	57 (82.609%)	5 (62.500%)
	Size range (bp)	219-227	219
	Na	4.000	1.000
	Ne	1.457	1.000
	I	0.608	0.000
	Ho	0.211	0.000
	He	0.314	0.000
DduB01	N	64 (92.754%)	6 (75.000%)
	Size range (bp)	320-370	326-346
	Na	22.000	3.000
	Ne	13.386	2.000
	I	2.748	0.868
	Ho	0.828	0.667
	He	0.925	0.500
DduB02	N	65 (94.203%)	8 (100%)

	Size range (bp)	218-244	218
	Na	5.000	1.000
	Ne	1.581	1.000
	I	0.765	0.000
	Ho	0.415	0.000
	He	0.367	0.000
DduCO5	N	62 (89.855%)	7 (87.500%)
	Size range (bp)	226-264	232-250
	Na	19.000	2.000
	Ne	11.81	2.000
	I	2.640	0.693
	Ho	0.823	1.000
	He	0.915	0.500

N=Number of sample, Na = observed number of alleles; Ne = effective number of alleles; I = Shannon's information index; Ho = observed heterozygosity; He = expected heterozygosity.
